# The use of food processing terminology in Australian news media: a content analysis

**DOI:** 10.1017/S1368980024000685

**Published:** 2024-04-01

**Authors:** Cherie Russell, Katherine E Sievert, Sarah Dickie, Priscila Pereira Machado

**Affiliations:** 1 School of Exercise and Nutrition Sciences, Deakin University, Geelong, VIC 3125, Australia; 2 Global Centre for Preventive Health and Nutrition, Deakin University, Geelong, VIC, Australia; 3 Healthy Food Systems Australia, Melbourne, VIC, Australia; 4 Department of Nutrition, Dietetics, and Food, Monash University, Melbourne, VIC, Australia; 5 Institute for Physical Activity and Nutrition, Deakin University, Geelong, VIC 3125, Australia

**Keywords:** Ultra-processed foods, Food processing, NOVA, News media

## Abstract

**Objective:**

This study aims to determine whether ultra-processed foods (UPFs) are being discussed in news media in Australia and whether this terminology, as described in the NOVA system, is being applied accurately.

**Design:**

Interpretive content analysis of online and print media articles that mentioned UPFs from 2009 to 2023 in Australia.

**Setting:**

Australia.

**Participants:**

Online and print media articles.

**Results:**

A total of two hundred ninety-eight Australian media articles were captured. A substantial increase in the number of UPF articles was observed between 2017–2019 and 2021–2023. The UPF concept was inaccurately explained or defined in 32 % of the articles and was frequently used interchangeably with other descriptors, such as ‘highly or heavily processed food’, ‘junk food’, ‘unhealthy food’, ‘packaged food’ and ‘discretionary food’. Most of the articles had a health focus; however, sustainability interest increased, particularly in the past 18 months.

**Conclusions:**

UPFs are increasingly being discussed in news media in Australia; however, the concept is still incorrectly presented in over a third of articles. This highlights the importance of improving the literacy about UPFs to ensure that messages are communicated in a way that is salient, accessible and accurate.

Poor diets, driven largely by our current dominant consumptogenic food system, are the primary contributor to the global burden of disease^(1)^. Defining poor diets has been a complex task due to differing views on the role of nutrients, foods and diets in human and planetary health^(2)^. Historically, diets were reduced to the sum of isolated nutrients existing in foods^(2)^. Thus, unhealthy diets have been described as diets deficient in essential vitamins, minerals, macronutrients or energy, or high in ‘risk’ nutrients (salt, saturated/trans fats and sugar) or energy^(3,4)^.

However, in 2009, the NOVA classification system was proposed based on the premise that, beyond nutrient composition, the extent and purpose of industrial food processing can adversely impact human health^(5)^. The NOVA system classifies foods into four groups: unprocessed and minimally processed foods, processed culinary ingredients, processed foods and ultra-processed foods (UPFs). UPFs are formulations of ingredients, mostly of exclusive industrial use, that result from a series of industrial processes^(6)^. These products are designed to be hyper-palatable, affordable and convenient^(6)^. They are often marketed intensively and are extremely profitable for highly concentrated transnational food corporations^(6–8)^.

The habitual consumption of UPFs is a marker of poor diets^(9)^ and has been associated with adverse health outcomes, including type two diabetes, CVD, cancer and all-cause mortality^(10)^. UPFs also have substantial environmental impacts associated with their production, such as high greenhouse gas emissions, deforestation, biodiversity loss, food waste, increased land conversion and excess water use^(11)^. In Australia, data from the most recent national nutrition survey (National Nutrition and Physical Activity Survey (NNPAS) 2011–2012) demonstrate that UPFs contribute 42 % of total energy intake^(12)^, ranging from 37 % among the elderly to 54 % among children and adolescents^(13)^. However, as UPF proliferation has increased over the last decade both globally and in Australasia^(14)^, this proportion has likely increased.

Despite its significance, research suggests that UPFs are not well understood or accurately communicated by academics, health professionals, advocates or the public^(15)^. In particular, there is evidence that transnational food corporations, who stand to lose the most from policy adoption of the NOVA terminology, have been deliberately misusing processing terminology as a way to dispute the evidence and cause confusion^(7)^. As the evidence of health, environmental and socioeconomical harms of UPFs increases, incorporating ultra-processing terminologies into the dialogue around health and sustainability is becoming a global policy priority^(16,17)^.

Media attention, measured by the volume of coverage an issue receives, is one way public health nutrition concepts and issues are communicated to populations, including in Australia^(18,19)^. Media articles provide a platform where the opinions, actions and statements of different policy actors converge^(20,21)^. Media attention is an important avenue for agenda setting and issue definition^(22,23)^ as heightened media coverage and salience of an issue increase the likelihood of capturing attention from policymakers, potentially strengthening political priority^(24–26)^.

Despite the increasing use of level of processing as a classification for foods within nutrition research and national guidelines^(27,28)^ and the increasing evidence of harms associated with UPFs^(9,10)^, no studies to date have demonstrated whether processing is being discussed in news media or how UPFs are framed. Thus, the aim of this study is to determine whether UPFs are being discussed in news media in Australia and whether this terminology as described in the NOVA system is being applied accurately.

## Methods

### Research design

We conducted an interpretive content analysis of Australian media to understand the extent of media coverage of UPFs in Australia.

### Data collection

We undertook a search of online and print articles that mentioned UPFs from both the Factiva and ProQuest databases. These databases contain a collection of news media sources from multiple disciplines in popular newsprint media available to Australian populations. Articles were included if published before May 2023 (the month of the search) and January 2009 (the first year NOVA was described in the peer-reviewed literature). We included all online and print news stories from these databases. Search terms included the following: ultra-process* OR ultra process* OR ultraprocess*. Mastheads included in the analysis reflect those in previous Australian media content analysis^(29)^ and capture a broad geographical range of Australian states and territories and their urban and rural areas.

Eligible articles were extracted from Factiva and ProQuest to Endnote X9. Duplicates were removed by A1. If the same article was replicated in multiple newspapers, only one instance was included in our analysis to prevent the misrepresentation of our results. Articles from the publication ‘NEWSRX’ were also excluded as these articles were reproductions of existing peer-reviewed journal articles, rather than news media reporting of these studies. Articles were then uploaded to the online screening and data extraction tool Covidence. The full text of each article was screened by A1 and A2 using the inclusion/exclusion criteria (Table 1). A second reviewer independently screened 25 % of the articles to minimise bias (A3). Disagreements were resolved through discussion and consensus within the research team.

**Table 1:** Inclusion and exclusion criteria

Inclusion criteria	Exclusion criteria
Published in English	Published in a language other than English
Published in an Australian newspaper	Letters, quizzes and magazine articles; articles not in Australian media
Published between January 2009 and May 2023	Published before 2009
Includes the word ‘ultra-processed’ correlated with health, diet or sustainability	Does not discuss ultra-processing in relation to health, diet or sustainability

### Data analysis

We systematically extracted details of each article to Microsoft Excel (V. 2112), including the author(s), year published, article title and publication, which terms were used to refer to processing, if the article refers to minimally or unprocessed food, if the article refers specifically to the NOVA system, if NOVA terminology has been used accurately, if other terms are used to describe UPFs (such as ‘junk food’), if UPFs are solely characterised by their nutrient or energy content alone (e.g. ‘UPFs are foods high in salt, sugar and fat’), if UPFs have been linked with a health or sustainability outcome, if the article is critical of NOVA or processing as a measure of healthfulness, the sector of any stakeholders quoted in each article, and characteristics of the included media outlets. For articles that made reference to different levels of processing, we used the NOVA framework^(6,30)^ as this is the most well-known framework to differentiate levels of processing and the authors are experts in the NOVA system. The NOVA food categories are defined in Table 2.

**Table 2:** NOVA categories for levels of processing (adapted from^(9)^)

Food category	Definition
(1) Unprocessed or minimally processed foods	Unprocessed (or natural) foods are the edible parts of plants (such as fruit, leaves, stems, seeds and roots) or from animals (such as muscle, offal, eggs and milk) and also fungi, algae and water, after separation from nature
(2) Processed culinary ingredients	Substances derived from group 1 foods or else from nature by processes such as pressing, refining, grinding, milling and drying. Examples include oils, butter, sugar and salt
(3) Processed foods	Foods made by adding salt, oil, sugar or other substances from group 2 to group 1 foods. Processes include various preservation or cooking methods. These include canned or bottled vegetables or legumes (pulses) preserved in brine; whole fruit preserved in syrup; tinned fish preserved in oil; some types of processed animal foods such as ham, bacon, pastrami and smoked fish; most freshly baked breads; and simple cheeses to which salt is added
(4) Ultra-processed foods	Formulations of ingredients, mostly of exclusive industrial use, typically created by a series of industrial techniques and processes (hence ‘ultra-processed’); carbonated soft drinks; sweet, fatty or salty packaged snacks; candies (confectionery); mass-produced packaged breads and buns, cookies (biscuits), pastries, cakes and cake mixes; margarine and other spreads; sweetened breakfast ‘cereals’ and fruit yoghurt and ‘energy’ drinks; pre-prepared meat, cheese, pasta and pizza dishes; poultry and fish ‘nuggets’ and ‘sticks’; sausages, burgers, hot dogs and other reconstituted meat products; powdered and packaged ‘instant’ soups, noodles and desserts; baby formula; and many other types of product

The coded data were used to identify major themes that were then synthesised in the results. We used an inductive content approach for our analysis, with the results discussed between the research team to limit researcher subjectivity^(31)^. We used Microsoft Excel to calculate descriptive statistics and generate graphical outputs.

## Results

**Fig. 1:**
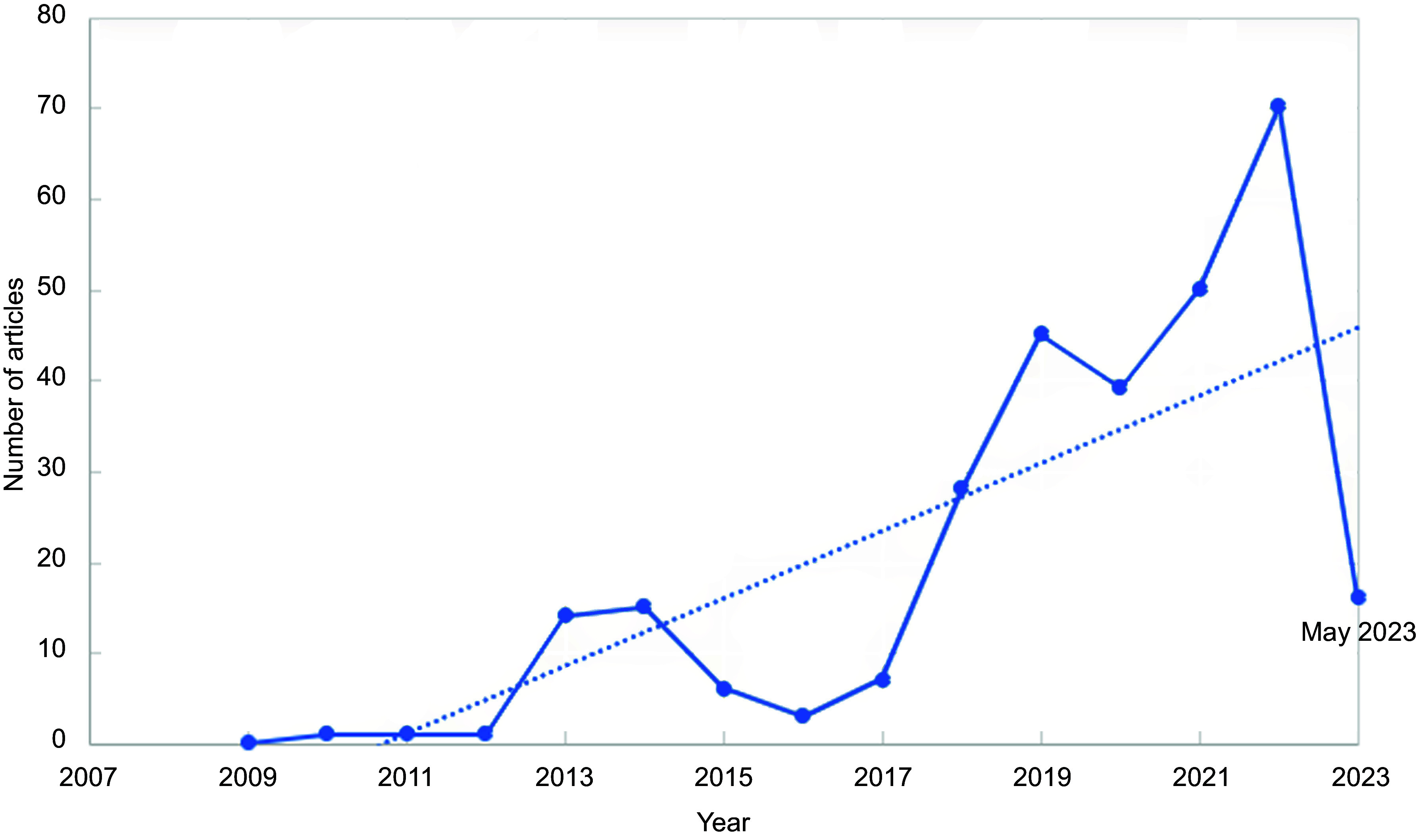
Frequency of Australian media articles published relating to ultra-processed food between January 2009 and May 2023 (two hundred ninety-eight)

We captured two hundred ninety-eight Australian media articles that discussed UPF in relation to health or sustainability in our search. The number of articles published increased over the period of analysis, with a substantial rise between 2017 and 2019 and from 2021 to 2023 (Fig. 1). A third of the articles were published in the last 18 months.

A total of eighty-three media sources contributed to the included articles. Table 3 shows the characteristics of the news media outlets. The majority of articles were derived from traditional print media outlets, with a subnational or regional focus, as opposed to tabloid (i.e. popular outlets smaller than the average broadsheet, largely comprised sensational stories) or subject-focused outlets (e.g. Agricultural News). Of the sample, 28 % of included outlets were ‘online only’, meaning no print version of the article was available.

**Table 3:** Characteristics of included media outlets (*n* 83)

Characteristics	Online-only publication	Online and print
Frequency of publication		
Daily	20	47
Weekly	3	12
Other (e.g. 4 d a week)	0	1
Region of focus		
International	10	3
National	9	15
Subnational/regional	4	42
Content focus		
News	12	40
Tabloid	2	12
Specific news (e.g. agricultural and economic)	9	8

In ninety-six articles (32 %), the concept of UPFs was inaccurately explained or defined. Examples included interchanging the term UPF with ‘processed food’ (i.e. foods that usually retain the basic formation of its original food structure but with added salt, oil, sugar or other substances^(9)^) (eighty-nine articles) and/or defining UPF by their nutrient and/or energy content (ninety-six articles). For example:

‘The major components of ultra-processed foods are sugar, refined carbohydrates, and seed oils’. (Article #285, quoting a doctor)

And

‘The best diet to boost your natural hyaluronic acid production is one free of processed foods and full of foods containing antioxidants (including vitamin C), zinc and magnesium’. (Article #238, quoting a nutritionist)

The term ‘ultra-processed’ was also used interchangeably with other descriptors, the most common of which included ‘highly or heavily processed food’ (sixty-four articles), ‘junk food’ (fifty articles), ‘unhealthy food’ (thirty-two articles), ‘packaged food’ (twenty-six articles) and ‘discretionary food’ (twelve articles). Few articles (fifty-four, 18 %) referred to minimally or unprocessed foods, while only three articles referenced the NOVA system specifically. No links were observed between the type of publication (news, tabloid or others), the frequency of publication or the region of focus and a tendency to conflate UPF with processed food or characterising UPFs by their nutrient or energy content. These conflations appeared to occur across the full range of included articles, no matter the media outlet.

UPFs were linked to several health outcomes in articles, including weight gain (obesity and overweight) (one hundred twenty-eight articles), type two diabetes (fifty-two), heart disease (forty-one), mental illness (mood changes, depression and anxiety) (twenty-seven), cancer (twenty-seven) and changes in the gut microbiome (eleven). Of the two hundred ninety-eight articles captured, one hundred twenty-nine articles quoted a stakeholder on the topic of food processing, most of whom were academics (sixty-nine) or health professionals (thirty-two). For example:

‘She said the majority of the population’s energy intake was instead coming from ultra-processed foods, which are highly detrimental to physical and mental health.’ (Article #66, quoting an academic)

A small majority of articles quoted only industry representatives or media personalities (nine articles), and these tended to be from tabloid publications or rural- and regional-focused outlets.

Most articles (two hundred fifty-four, 85 %) did not challenge the concept of level of processing as a measure of healthfulness. Those articles that did present a critique of the NOVA concept either quoted a stakeholder from the food industry or were written by an author with financial ties to the food industry. Only three articles were explicitly focused on disputing the use of processing as a measure of healthfulness. These articles defended the food industry and urged for the ‘demonisation’ of incorrectly defined ‘processed foods’, rather than UPFs, to cease. For example:

‘There is no sound scientific validation for linking all processed food to obesity. In contrast, diets high in—e.g. high in energy, sugar, or fat—may contribute to weight gain and obesity’. (Article #274, quoting a spokesperson from Nestle)

Of the three, two articles were written by academics, both with ties to food industry funding. The remaining article was written by a business journalist in a food industry publication. Two of these articles quoted a food industry representative. One article from a national news outlet, quoting an academic, appeared to identify this industry strategy as part of a broader suite of tactics to discredit public health efforts to regulate UPFs.

‘The ultra-processed food industry undermine virtually every public health proposal that is put forward,’ he says. ‘The only thing they are interested in is utterly ineffective self-regulation’. (Article #23)

Only twelve articles linked UPFs to environmental sustainability outcomes, ten of which were published after 2021. The most prominent outcomes were related to climate change, greenhouse gas emissions, food waste and packaging. Around half of these articles were discussing ultra-processed meat alternatives and their environmental and health impacts relative to less processed animal source foods. For example:

‘The misperception that “alternatives” are more planet-friendly has not only been driven by the animal rights lobby but by big food corporations who have identified “just another great market for processed food”’. (Article #170, quoting an academic)

## Discussion

This study aimed to determine whether UPFs are being discussed in news media in Australia and whether this terminology, as described in the NOVA system, is being applied accurately. We found that UPFs are being increasingly discussed in the Australian media, particularly since 2017. The increased attention might be explained by the publication of several high-quality prospective cohort studies in Australia and internationally and a randomised controlled trial^(10,32)^, which have strengthened the evidence of UPFs and adverse health outcomes^(9)^. A relative drop in UPF coverage was observed in 2020, which may be related to the global pandemic of coronavirus disease 2019 (COVID-19) that dominated news coverage during that period. Our results seem to indicate an interest in UPFs across the spectrum of news media outlet types, including national and subnational outlets, online and print, and news and tabloid media types. There is limited evidence on how UPFs are interpreted by different socioeconomic, age and sex demographics; however, these data were not available for all media outlets included in the sample and so were not analysed. The widespread coverage of the UPF concept, particularly over the last 2 years, seems to indicate it is of interest to readers. Future research into differentials among readership demographics may be of benefit for a more nuanced understanding.

Nutrition science has been traditionally nutrient-centred and unhealthy foods commonly defined by the presence of harmful nutrients^(3,27,28)^. UPF is a relatively novel concept (first presented in 2009) and not well understood among consumers and some nutritionists and food scientists^(15)^. We found that the UPF concept was inaccurately explained or defined in one third of the articles. They were frequently defined as being unhealthy due to their energy density or high content of nutrients of concern (such as sugar or fat), rather than as a result of processing itself. Importantly, although UPFs do tend to be high in energy and nutrients of concern, associations between higher consumption of UPFs and health outcomes remain after adjustment for nutrient content^(33)^.

Moreover, the UPF term was often substituted with terms such as ‘highly or heavily processed food’ or ‘junk food’, yet UPFs include many foods perceived to be healthy (such as some breakfast cereals or plant-based milk), which can contribute to public confusion. Interchanging UPF with the term ‘processed food’ particularly is unhelpful since almost all foods undergo some level of industrial processing, and some types of food processing can contribute to healthy diets (freshly baked breads, cheeses and canned vegetables, legumes and fish) where others may be harmful (confectionery, fast food, potato crisps and protein bars). Due to its ability to differentiate foods based on the purpose and extent of industrial processing, the NOVA system specifically identifies UPFs as a class of foods that are unnecessary in a healthy diet for the general population^(6)^. It is acknowledged that certain clinical conditions and individual circumstances, such as food intolerances and the requirement for infant formula, present exceptions in which UPFs may be a necessary inclusion in the diet^(6)^.

Nonetheless, the UPF concept is also a political one as it challenges the power of corporations that produce these foods^(34)^. Transnational corporations, who stand to lose the most by adoption of the NOVA terminology in public health policy, have been deliberately misusing processing terminology as a means to dispute the evidence and cause confusion, particularly for consumers. For example, some food industry groups have attempted to undermine the increasing evidence base associating UPF with poor health outcomes by referring to group 3 processing and conflating it with ultra-processing techniques^(35,36)^. Attempts to sow confusion have also been observed among academics with food industry links reporting to the media^(37)^. This is most illustrative in the results of this analysis whereby the articles that explicitly discredited ultra-processing as a marker of healthfulness were in some ways connected to the food industry. Allowing the food industry to discursively influence how these issues are raised allows for a favourable policy environment that does not challenge their power. These tactics have been observed in other fora, including at the 2021 United Nations Food Systems Summit, whereby the private sector was the dominant participants and set the agenda for much of the discussions^(16,38)^.

Other potential reasons why the UPF concept was applied incorrectly or interchangeably with other terms may be a result of poor translation from academia to journalism. Journalists tend to use synonyms or terms with same connotations throughout an article to avoid being repetitive or to facilitate readability. To illustrate, journalists often use ‘climate change’ and ‘global warming’ interchangeably, despite the former being the appropriate scientific technical term, with global warming as one of its components^(39)^. The misuse of the term has contributed to public confusion, increased polarisation, weakens public support and political inaction on climate issues^(40)^. Similarly, technical integrity in the communication of UPFs is key to avoid the concept being misconstrued by the public and misinformation to be repeated and amplified by the media.

The significant increase in UPF coverage in recent years in Australia reflects the growing interest of the public on this issue and its relevance in current public policy developments^(41)^. There is a momentum to respond to the human and planetary harms associated with increased consumption of UPFs in Australia; thus, addressing the challenges of UPF communication is critical. Mass media coverage plays an active, crucial role in shaping both dietary behaviours and policy agenda setting, by influencing the public and policymakers’ perceptions of an issue. Increasing awareness of UPF harms through appropriate media framing has the potential to generate traction for public support for policies targeting UPFs, while also increasing nutrition literacy of the population to reduce UPF consumption. Lessons from the COVID-19 pandemic and climate change (mis)information put science communication and the need for health literacy at the centre for addressing pressing health challenges^(40,42)^.

The NOVA system, despite serving as a valuable tool for measuring and monitoring poor diets, is not exempt from critique^(43)^. The wide range and heterogeneous selection of the food supply covered by the UPF definition have been a point of contention^(44)^. Moreover, the proliferation of UPF, particularly in ‘food swamps’ in low socioeconomic areas, serves as significant social and economic barriers to limiting the ability to reduce UPF consumption^(45,46)^. Furthermore, a prevalent concern in the literature is that UPFs provide a substantial proportion of daily nutrients to low socioeconomic populations, and discouraging their consumption may lead to nutrient deficiencies in the population^(47)^. However, this argument assumes UPFs would be eliminated from diets without the substitution of nutrient-dense non-UPFs. These arguments also fail to consider the wider burden of chronic disease risks in those who are more food insecure and for those whose food budgets are restricted^(48)^. Despite concerns with the concept, NOVA is still widely recognised for its capacity to identify poor diets, and it is the classification system based on food processing most applied in research and policy worldwide^(9)^.

There is a need for more consensus on terminology around processing for public policy to effectively move forward in addressing poor diets. This includes establishing clear definitions and guidelines regarding UPFs^(28)^, especially in national policy documents such as the Australian Dietary Guidelines as is being done in other national guidelines around the world^(27,28)^. Given the inconsistencies and confusion regarding the NOVA categories of ‘processed’ and ‘ultra-processed’ foods, there is a need to ensure that these concepts are communicated to both professionals and the community in a way that is salient, accessible and accurate. It is important that this term is clearly understood given the confusion and contention around processing as a measure of healthfulness. There is evidence that simple educational interventions using NOVA classification principles are easier to understand and apply compared to the common principles of food groups and ‘nutrients to limit’ present in dietary guidelines^(49)^. Furthermore, providing scientific training for journalists on this concept to critique the research accurately, as exemplified by initiatives in Brazil^(50)^, can contribute to reducing miscommunication and promote more accurate reporting to ultimately raise awareness.

### Conclusion

The use of level of processing to describe foods, dietary patterns and broader health outcomes is on the rise, though the concept is not always applied correctly. In Australian media, the UPF concept is not accurately reported, which has effects on public understanding. Promoting a greater understanding of the concept among academics, advocates, policymakers and the public regarding what UPFs are, their implications for health and environmental sustainability and how they differ from other types of processed foods will be vital in developing and achieving meaningful policy change to improve human and planetary health. This is particularly important in the context of food industry spokespeople attempting to discredit the UPF concept, given that it facilitates more comprehensive discussions beyond mere nutrient content, including topics related to sustainability and corporate power.
